# Do rats learn conditional independence?

**DOI:** 10.1098/rsos.160994

**Published:** 2017-02-08

**Authors:** Robert Ian Bowers, William Timberlake

**Affiliations:** 1 Cognitive Science, Indiana University, Bloomington, IN, USA; 2 Psychological and Brain Sciences, Indiana University, Bloomington, IN, USA; 3 School of Psychology, Universidade do Minho, Campus de Gualtar, 4710-057 Braga, Portugal

**Keywords:** *Rattus norvegicus*, conditionality, causal reasoning, associative learning, graphical models, Markov condition

## Abstract

If acquired associations are to accurately represent real relevance relations, there is motivation for the hypothesis that learning will, in some circumstances, be more appropriately modelled, not as direct dependence, but as conditional independence. In a serial compound conditioning experiment, two groups of rats were presented with a conditioned stimulus (CS1) that imperfectly (50%) predicted food, and was itself imperfectly predicted by a CS2. Groups differed in the proportion of CS2 presentations that were ultimately followed by food (25% versus 75%). Thus, the information presented regarding the relevance of CS2 to food was ambiguous between direct dependence and conditional independence (given CS1). If rats learnt that food was conditionally independent of CS2, given CS1, subjects of both groups should thereafter respond similarly to CS2 alone. Contrary to the conditionality hypothesis, subjects attended to the direct food predictability of CS2, suggesting that rats treat even distal stimuli in a CS sequence as immediately relevant to food, not conditional on an intermediate stimulus. These results urge caution in representing indirect associations as conditional associations, accentuate the theoretical weight of the Markov condition in graphical models, and challenge theories to articulate the conditions under which animals are expected to learn conditional associations, if ever.

## 1. Introduction

Covariation observed in the world may be owing to direct relevance between covarying events, such as one causing the other, or to any of a number of indirect relations. Contemporary theory on learning motivates drawing such distinctions, and yet the question of whether animals in conditioning experiments learn differently about direct and indirect relevance remains. Consider the case in which a tangible correlation exists between non-adjacent stimuli in a stimulus chain. If the intermediate stimulus is consistently present, then the information is ambiguous between two possible relevance structures: the observed covariation between the non-neighbouring stimuli may indicate direct dependence or independence conditional on the intermediate stimulus. Do animals distinguish these two possibilities?

The question is motivated by consideration of the structure of networks. By a range of perspectives on learning, what animals are expected to learn about predictive relations among stimuli in conditioning experiments may be described as a network of associations. This is true even of the staunchly quantitative approach typical of theories of associative learning [1–3], although by such views, all of the information visualized as a network can be captured by a list of association strengths, which might be separately represented by the learner (as opposed to being represented together in a coherent network model). An exception is seen with configural-cue theories [4], in which networks also represent hypothesized hierarchical relations among elements. Other perspectives, in contrast, expect qualitative variety in the structure of what animals learn. These include behaviour systems views [5–7], which focus on problem-specificity and species-typicality, as well as graphical model-based theories [8,9], such as Bayesian networks [10], which conversely provide analyses in general, abstract terms. Network diagrams that appear in behaviour systems theorising have tended to be limited to representing isolated aspects, such as hierarchical relations [11], and the perspective has so far borrowed little expressly from network thinking. A strength of graphical model-based theories is their use of available theory from mathematics to put to work this image of a network. Doing so provides an additional source of insight regarding diversity of structure in learning.

Conditional^1^ relevance is one crucial aspect upon which network structures may vary, and marks a central distinction between graphical model-based and association strength-based views. Events may be associated directly (first-order), or indirectly, via an intermediate event (second-order) or set of events (higher-order). Second-order conditioning^2^ is well documented in rats [12–14], but a second-order association is not necessarily a conditional association. If A is associated with B, and B is associated with C, by some views, but not others, the relationship between A and C is conditional on knowledge of the current status of B. For instance, if, for a laboratory pigeon, appearance of the experimenter (A) often precedes a training session (B), and this involves being fed (C), there is a real relationship between the appearance of a white coat and receipt of food. However, one's ability to predict the latter from the former is conditional on what is known about the intermediate event; once the pigeon is on its way to the Skinner box, the prediction about receiving food is no longer aided by presence of the white coat. In this case, the relationship appears to be conditional.

However, dominant views do not treat second-order associations as conditional associations. This qualitative distinction is not drawn, and the difference between first- and second-order associations is viewed in exclusively quantitative terms, and so is their effect on behaviour. Pavlov's [15] own view of second-order conditioning is understandable in the light of his theory of stimulus substitution, which posits that a conditioned stimulus (CS) becomes a surrogate for the unconditioned stimulus (US). In second-order conditioning, this surrogacy is passed on from first-order to second-order CSs. Hence, the second-order CS is just a fainter shadow of the US. Although stimulus substitution has been rejected with some confidence [16], the core of Pavlov's thinking still exerts considerable theoretical inertia. By the even older and equally influential ‘association of ideas’ tradition of Locke and Hume [17], the second-order cue ‘brings to mind’ the first-order cue, which brings to mind the US. Again, the ‘idea’ of the second-order cue is just fainter than the first-order. Neither of these perspectives inclines one to view a second-order association as a conditional relationship. Importantly, both the surrogacy of the former, and the ‘bringing to mind’ of the latter, bypass any requirement for knowledge about the status of the intermediate stimulus, as would be the case for a conditional association. Note that although second-order conditioning has been amply documented, the empirical evidence for higher-order conditioning has been spottier and less prolific than expected, especially given the motivation to extend associative theory to complex learning [18]. If these associations are conditional, however, a third- or fourth-order association might manifest no less strongly, but only under very specific circumstances, which an accurate theory would be expected to anticipate.

Among graphical model-based approaches, the direct dependence or independence of each pair of events is explicitly modelled, and if two nodes are linked only via an intermediate node, this indicates a conditional association. This is articulated as the Markov condition [8,10]: the requirement that any node in a network is independent of its non-neighbours, conditional on its neighbours. (For a directed (e.g. causal) network, the constraint is directional: every node is conditionally independent of its non-descendants, given its immediate antecedents). This means that one need only consider the local relevance environment of an event to assess its likelihood, not the predictors of predictors, and so forth. This provides a great computational shortcut, making a complex web of interrelated events more feasible to navigate. Given this assumption, the probability of an event occurring can be calculated without knowing much of the network. Consider how difficult it would be to predict the weather in Bloomington, for instance, if one needed to consider not only the current weather in the immediate area, but also all of the relevant factors behind that—the weather on the continent over the last fortnight—and everything that predicted that, and so forth, down to what impact the movement of a particular whale had on local currents as it passed the doldrums (or wing beats of migrating butterflies in Japan). As a network becomes more complex, if every eventuality needs to be considered, the problem of calculating the probability of an event quickly becomes practically intractable. Assuming the Markov condition allows such probabilities to be calculated given knowledge only of immediately relevant events. Without conditionality, there are no such shortcuts.

The Markov condition furthermore fits intuitions about relevance. For instance, a mosquito may buzz audibly with some probability. The probability of hearing a mosquito's buzz in the room depends on a large number of factors, including humidity, time of year, proximity to water, whether windows are open, whether there are screens in the windows, whether there are holes in the screens, whether the room is in a forest, or plains, or a city, whether the city applies insecticides, etc. If all of these facts about the world impact the probability of hearing a mosquito's buzz, it would seem impossible to calculate. However, once the immediate source of the mosquito's buzz is identified, and the errant mosquito is spotted, all of the factors that contribute to the mosquito being present or absent fade from relevance. For assessing the likelihood of hearing the buzz, it should no longer matter how probable a mosquito's presence is under normal circumstances, just that it is there. The Markov condition accords precisely with this intuition.

**Figure 1.**
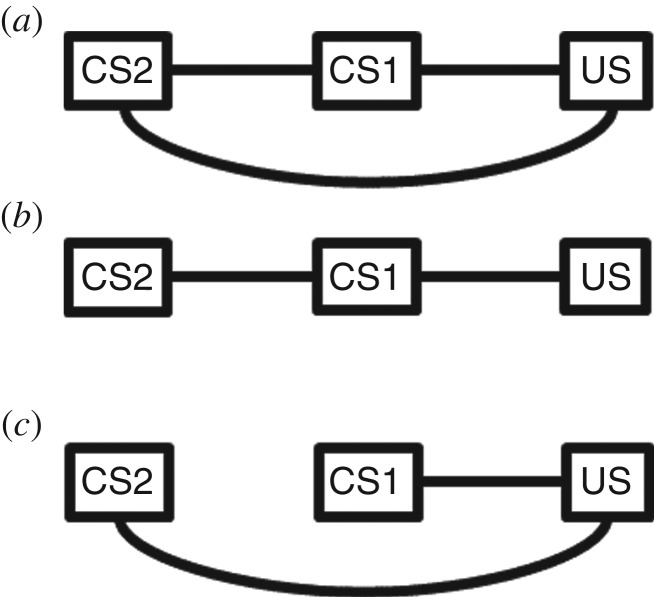
(*a*–*c*) Three network structures that predict responding to CS2, any of which may feasibly be acquired with serial compound conditioning (CS2, followed by CS1, followed by US). The analysis applies whether the edges are directed (left to right) or undirected.

The present work focuses on the simple case of serial compound conditioning: a sequence of two cues (CS2 followed by CS1) is followed by a food US. Such training produces experience-dependent responding to CS2 [19], which is consistent with, either, a direct or a conditional association between CS2 and food. Views that assume the Markov condition are obliged to represent these two possibilities differently, differentiated by the presence or absence of a first-order association between CS2 and food. We can thus cast the question in such terms, as uncertainty between the following two feasible network structures: a fully connected network in which CS1, CS2, and food are all directly interdependent (as in figure 1*a*), and a sparser network in which CS2's association with food is only second-order, via CS1 (as in figure 1*b*). In the former case, associations directly link all the events, and so satisfying the Markov condition is trivial. If the latter, the Markov condition stipulates the following: given the immediate antecedent of food (CS1), its antecedent (CS2) is independent of the appearance of food. This reference to graphical models is pragmatic, to borrow the clarity the perspective provides regarding how the presence or absence of a first-order association is expected to affect behaviour.

Although both hypothetical networks permit the prediction of US from CS2, the fully connected network (figure 1*a*) permits no shortcuts. The sparser model in figure 1*b* benefits the animal in the same ways that the Markov condition applied to conditional associations benefits the engineer using graphical models to calculate conditional probabilities. First, the task of making decisions with sparser graphs is easier (and so faster, and potentially more accurate under time or processing constraints). Second, an incompletely connected graph may often fit the actual relevance relations more accurately than the purely quantitative discrimination available in the alternative. Third, although this distinction may seem of little practical consequence in the simple case presented, sensitivity to the difference between direct and conditional associations scales up to provide a basis for acquisition of network structures of greater complexity and variety.

A completely connected network with edges between every pair of nodes in a complex web of interrelated events may exhibit much redundancy, as first-order associations are drawn between events that are also associated by higher-order paths. Omitting redundant links not only yields a more economical model, but also produces grounds for qualitative diversity of network structure. A fully connected network, in contrast, will not exhibit complexity or variety of structure beyond the purview of association strength-based theories. Such structural distinctions delimit how inferences can be drawn among non-adjacent events [10], bringing potentially subtle but tangible implications for behaviour. This accentuates the centrality of conditional associations for assessing the potential value of appealing to theories that concern network structure for analysis of associative learning. If the graphical model theorist is inclined to model learning with fully connected networks, the approach loses an aspect that motivates it, a source of insights absent in previous approaches.

The present study tests a ‘conditionality hypothesis’ that animals will learn a conditional association when the information presented warrants. In the case at hand, CS2 never signals food alone; CS1 precedes every arrival of food. Thus, even though CS2 and food covary, the information presented is ambiguous between a relation of direct relevance and one that is conditional on CS1. Which of these two kinds of relevance relations will the subjects learn? These possibilities can be discerned experimentally. In particular, by the conditionality hypothesis (represented in figure 1*b*), when CS1 is present, the presence or absence of CS2 should have no impact on expectation of food, and because CS1 is consistently present on acquisition trials, the vigour of the conditioned response (CR) acquired to CS2 should depend on the strength of CS2–CS1 and CS1–food associations, and be impervious to manipulations of the degree to which CS2 and food themselves covary.

Among graphical model-based theories, a common approach to learning conditional associations can be traced to treating training contingencies as if they were probabilities. Conditional independence of probabilities has a formal definition. As probabilities A and C are conditionally independent given B just when P(C|A,B) = P(C|B) (or equivalently, when P(A,C|B) = P(A|B) P(C|B)), some graphical model-based theories [20] predict that learners will form conditional associations just when the proportions of stimulus pairings follow the analogous rule. Thus, by such views, only if the proportion of US presentations, given CS1, is the same, regardless of whether CS2 has occurred or not, should the learner acquire a conditional association between CS2 and US, via CS1. Although we caution against uncritical slippage between talk of contingencies and talk of probabilities, the connection is neither new nor without support [21].

Single-effect learning theory (SELT; [22]) is a graphical model-based theory of learning that takes an especially clear stance on the present question. By SELT, the ‘common-effect’ structure is the basic unit of learning. In a given training trial, the animal attends only to one potential effect of any apparent cause. Alternative causes, observed or otherwise, affect learning in the trial, but other potential effects of these causes are transiently ignored. Waldmann *et al*. [22] stress that SELT does not require the animal to represent coherent network models of learnt associations. Rather, each effect with its direct causes is assumed to be learnt and represented separately. This enforces conditional independence. Adherence to the Markov condition in SELT is, thus, ‘a side effect of chaining the inferences’ [22, p. 462]. In the case of serial compound conditioning (CS2, then CS1, then food), formation of a CS1–food association is uncontroversial. SELT predicts that CS2 will acquire association with only one potential effect; CS2 is predicted to become associated with either CS1 or food, but not both.

The conditionality question is less obvious from an associationist perspective because of a precedent set, again, by Pavlov in ascribing a central role to the unconditioned in associative learning. He characterized the acquisition of response propensities of initially neutral stimuli as involving association with, in principle, a stimulus that evokes responding independently of experience. Whereas earlier views of association made no such assumptions [17] and later thinkers challenged them (concisely reviewed in [23]), the primacy of the unconditioned in associative learning was a cornerstone of behaviourism, embedded in the very language of learning research. It is present even among newer theories [3], and retains considerable theoretical inertia today. This impacts the current question, because the standard view has all CSs anchored on the US. Thus, when Holland & Ross [19] explored the associations formed among stimuli in a serial compound conditioning procedure, direct associations were assumed to form between food and each CS (as in figure 1*c*). In that theoretical climate, the controversial association in need of empirical support, and of especial relevance to contemporary theories focusing on stimulus interactions, was the CS2–CS1 association. This is quite unlike how inspiration by graphical model-based theories inclines one to draw links among these events, by which the model in figure 1*c* is less intuitive. The outcome of Holland & Ross [19] and other research and theorising of the era was that associations do form among CSs, ultimately leaving the field with a consensual picture of associations no longer like figure 1*c*, but like figure 1*a*, a fully connected network.

Behaviour systems approaches have likewise inclined proponents to view all components of a complex CS (i.e. both CS2 and CS1, in the present case) as having direct relevance with the unconditioned [24]. However, by such views, the unconditioned is not a discrete event or stimulus, as association strength-based and graphical model-based views have tended to approach it, but a motivational system. Learning consists of fitting patterns of occurrences in the external world, to the malleable but persuasive structure of the internal. By such views, earlier and later CS components are expected to connect with the same system, but different parts (‘modes’) of this system. Thus, a behaviour systems perspective remains distinct as a third position.

### 1.1. Review of previous relevant research and theory

Issues of relevance to the current question of conditionality have come up before in different theoretical contexts. Although the motives were different, such work is helpful to consider in moving forward. Note that some of the recurrent names in the ensuing discussion straddled the sides on the current question. It would be anachronistic to expect these workers to fit into current camps, or to give a simple or singular answer on the question of conditionality, as they were tackling different problems, in different theoretical and historical contexts.

#### 1.1.1. Cue competition

Throughout the 1970s and 1980s, a bouquet of new theories of associative learning emerged to account for cue competition effects, a growing body of evidence suggesting interdependence among multiple covarying cues in conditioning circumstances, e.g. [25]. In the Rescorla-Wagner model [3], for instance, assumption of dependence among CSs is central: ‘the effect of a reinforcement or non-reinforcement in changing the associative strength of a stimulus depends upon the existing associative strength, not only of that stimulus, but also of other stimuli concurrently present’ [3, p. 73]. This assumption is shared by theories focusing on temporal factors (e.g. relative waiting time hypothesis: [26]), as well as attentional models [27,28], and configural-cue approaches [4]. Similarly, associative theories concerning performance also stress dependence among focal and contextual stimuli (e.g. comparator hypothesis: [29]). Although the dependence between competing predictors of a US acknowledged by such views is not presented as conditional on the status of the US, there is no suggestion that learners associate competing cues in other circumstances. Rather, the dependence is presented as specifically concerning learning about the CS–US relationship. Note that this comes very close to a claim about conditionality.

Cue competition is readily interpretable in terms of conditional relevance. If stimulus A and stimulus B each predict food, even assuming that they otherwise occur independently of each other, they will be related conditionally: the appearance of food heightens the likelihood of both predictors (A and B) above baseline, and in this condition of heightened likelihood, A and B become inversely related, because the presence of any one predictor, lessens expectation of any other. Hence, cues compete. Formally, this circumstance is analogous to the ‘common effect’ structure, and is a good example of a specific manner of conditional relevance expected between otherwise independent events [10]. By this interpretation, what is general about cue competition is not reinforcement, but conditionality.

#### 1.1.2. Stimulus–response learning and devaluation

In a purely stimulus–response (S–R) machine, the CS directly evokes the CR, without reliance on internal representation of any kind, including goals, or a path of intermediate associations. By such a mechanism, all that is registered is how to respond under specific stimulus circumstances. There are no grounds for differentiating discrete, intermediate associations, and therefore, no grounds for conditionality. Hence, evidence of S–R learning suggests absence of conditional associations, and evidence of intermediate representations establishes a necessary condition for conditionality.

Advances in learning theory have addressed questions concerning S–R learning with studies of US or outcome devaluation. Satiation or induced illness can devalue a food reinforcer. Pavlovian [30] and instrumental [31] conditioning studies with food have shown that devaluing food after conditioning attenuates food-related responding to the CS or manipulandum subsequently presented alone. Such results encourage the view that subjects form a representation of the food, which mediates stimulus and response, and discourages interpretations in terms of a direct S-R association, by which the CR should not be affected by devaluation.

Of additional relevance to the question of conditionality, Holland & Rescorla [30] furthermore showed that the impact of US devaluation on responding during extinction to a first-order CS, was not observed to a second-order CS. Similarly, Holland & Rescorla [14] showed that following second-order conditioning, extinction training with the first-order CS had no effect on experience-dependent responding to the second-order CS. In both cases, responding to the second-order CS was independent of the manipulation affecting responding to the first-order CS. Both results appear to suggest that the second-order association was not conditional. One interpretation of such results is that second-order conditioned responding relies more heavily than first-order conditioned responding on an S–R mechanism [32]. In either case, second-order learning appears to be unlike first-order learning. These two observations push in opposite directions on the present question.

#### 1.1.3. Serial compound conditioning

Holland [13] assumed that serial compound conditioning with a two-element CS produces, both, a direct, first-order association of the first stimulus (CS2) with the US, and a second-order association via CS1. On the assumption that devaluation interferes with expression of first-order but not second-order associations [30], devaluation by satiation was used to dissociate these two kinds of effect on experience-dependent responding to CS2. Holland [13] showed that training manipulations predicted to differentially interfere with second-order versus first-order conditioning produced reduced experience-dependent responding to CS2 relative to controls when subjects were sated (assumed to primarily reflect second-order conditioning), but largely the converse was observed when the same subjects were subsequently tested under standard food deprivation (assumed to primarily reflect first-order conditioning). Although the predictions Holland [13] was testing, concerning the differential effects of ‘surprise’ on first-order and second-order conditioning, are orthogonal to the current question, the success of his approach of assuming both first- and second-order associations provides indirect evidence against the conditionality hypothesis.

After serial compound conditioning with a light–tone–food sequence, Holland & Ross [19] showed that subsequent response to either stimulus in the series was attenuated by presenting the other stimulus alone in an extinction phase. Both results suggest the presence of a stimulus–stimulus association between the tone and light mediating experience-dependent responding to either. However, not all CR forms were affected by the extinction procedures, including nosing of the food trough. The absence of such an effect following extinction of the second stimulus suggested to Holland & Ross [19] either maintenance of an S–R association, or, relevant to the current discussion, presence of a direct association between the first stimulus and food.

#### 1.1.4. Second-order to inhibitory conditioning

Yin *et al.* [12] noted similarities between second-order and inhibitory conditioning procedures. Both involve separate CS1–US and CS2–CS1 pairings, and thus present a conflict of information about the relationship between CS2 and US: despite a positive second-order relationship via CS1, there is also a negative relationship, as CS2 signals the absence of the US. If subjects represent the positive, indirect relationship, such training should produce excitatory responding to CS2. If the negative, direct relationship is represented, an inhibitory pattern is expected. The predictions are opposite depending on whether subjects acquire the indirect or direct association. There is a rich literature of both patterns obtaining, in second-order conditioning, and inhibitory conditioning, respectively.

Using a second-order conditioning procedure that produced substantial excitatory responding to the second-order CS2 with few CS2–CS1 trials, Yin *et al.* [12] showed that with continued training (while keeping the number of CS1–US trials constant), CS2 acquired inhibitory status. Excitatory responding, such as was observed early in the experiment, is expected only if the represented relation is conditional; development of an inhibitory pattern suggests direct dependence. Given the important role of experience with CS1 in changing the valence of the response to CS2, the CS2 and US appear not to be independent after extensive training, although earlier in training, they might have been. This can be taken as evidence that the amount of training is relevant to whether conditionality holds or not. This change in valence with continued, consistent evidence for an association presents an empirical challenge that may provide an important future battleground, as it pushes association strength-based and graphical model-based theories to different solutions.

### 1.2. The experiment

In the present experiment, a rolling ball bearing (BB; CS1) is followed by food half of the time, and a neutral light stimulus the other half of the time. Half of these presentations are preceded by insertion of a lever (CS2). The question concerns what the rat learns about the lever–food relation in this circumstance. The lever will become an excitatory stimulus [19], and lever contact is expected to develop, but this may be owing to a first-order lever–food association, or a second-order association via CS1. Does the rat learn that the lever is a predictor of food, or only a predictor of a predictor of food? To assess this, we varied the reliability with which the lever predicted food, while keeping other relations constant. In one group, the lever was followed ultimately by food most of the time (75%), and in the other, only a quarter of the time. Meanwhile, the quantity of lever–CS1 and CS1–food pairings were the same in both groups (table 1). In the test phase, the lever was inserted alone.

**Table 1.** Training procedure (number of presentations of each type).

	group 75%+	group 25%+
lever–CS1–food	15	5
CS1–food	5	15
lever–CS1–light	5	15
CS1–light	15	5

If what the subjects learn about the relation between CS2 and food is only that CS2 predicts CS1, and CS1 predicts food (conditionality hypothesis), the intensity of feeding-related responding to CS2 presented alone in the test phase should not depend on the extent that CS2 varied with food in the training phase, which differed between groups, but expressly on the extent that CS2 varied with CS1, and CS1 varied with food, which were the same in both groups. Thus, both groups should respond to the lever in the test phase similarly. By contrast, if subjects learn a direct, first-order CS2–food association, heartier responding to the lever should be shown by the group for which the lever was more often followed by food.

The presented information is ambiguous between a direct and a conditional relationship. While the consistent order of stimuli in the training phase encourage learning the second-order association, the tangible concurrence of CS2 with food provides statistical evidence that CS2 and food are dependent, though not whether this dependence is conditional or direct. Which will the subjects acquire? Theories are unsurprisingly divided on the stress each places on statistical versus other sources of evidence for deciding the content of learning. Association strength-based theories [1–3] are joined by a large subset of graphical model-based theories in their strong (though not exclusive) focus on statistical information [20,34]. These will be inclined to agree in predicting that subjects will be moved by the statistical evidence for a direct dependence of food on CS2, and form a direct, first-order association between them. Other graphical model-based theories may be uncommitted [35], whereas others are oppositely inclined [22].

SELT [22] is one graphical model-based theory of learning that clearly predicts the conditionality hypothesis. By SELT, our subjects should come to associate only one consequent stimulus with CS2. Given that CS1 follows every presentation of CS2, and immediately, SELT predicts acquisition of a CS2–CS1 association, and the relation between CS2 and food to go unnoticed. According to SELT, subjects should represent this CS2–CS1 association, and the uncontroversial CS1–food association, separately.

## 2. Methods

### 2.1. Subjects

Twelve female Sprague–Dawley rats served as subjects. For the course of the experiment, they were housed individually on a 12 h light : dark schedule. They were fed once daily, 1–3 h after dark, and maintained at a minimum of 85% of their free-feeding weights. Water was freely available in home cages.

### 2.2. Apparatus

The conditioning chambers were made of sheet metal (50 × 35.5 × 30 cm), accessed by a hinged Plexiglas front wall. These were in one room, and linked to data-logging and control equipment in an adjacent room. The food trough (3.1 cm wide, 5.9 cm high, 1.8 cm deep, 4 cm above the floor) detected intrusions by breaks in an infrared beam that projected across its entrance. Food delivery consisted of two 45 mg Testdiet purified pellets released into the feeder (1 s apart). BBs (1.6 cm diameter) entered the chamber via a hole in the far wall, rolled along a recessed track in the floor (2.1 cm wide) lined by two parallel steel rods that ran the length of the chamber (6° slant; 7.2 cm from the front wall), and exited through a second hole on the feeder wall. If undisturbed, once released, the BB takes approximately 5 s to exit. The retractable lever (3.6 cm wide; 4 cm above the floor) was immediately above the BB exit hole. The speaker (centred 1.6 cm above the lever) emitted a 1440 Hz tone. A weight-sensitive platform in the floor of each chamber (extending 10.2 cm from the wall, 12.5 cm wide) provided a measure of when the subject was immediately before the food trough.

### 2.3. Procedure

For each of the 5 days of the training phase, subjects received eight presentations of a rolling BB stimulus at variable intervals (mean: 4.7 min; minimum: 3 min) over a 42.4 min session. After 5 s (roughly the amount of time it takes for the BB to exit if unperturbed), the BB was followed by either food (50%) or a 5 s tone (50%). Half of the BB presentations were preceded by insertion of a lever into the chamber. The lever would retract at the moment of BB release, which was after the passage of 30 s, or when the lever was contacted by the rat (except within the first 5 s, during which lever contact had no such effect, to assure 5 s of measurement).

In group 75%+, food followed 15 out of 20 of lever-cued BB presentations, but only 5 out of 20 of uncued presentations. In group 25%+, food followed 5 out of 20 of lever-cued BB presentations, and 15 out of 20 of uncued presentations. Each training session consisted of four lever-cued and four uncued presentations at variable intervals.

An extinction test was conducted for five further days. On day 6, the lever was inserted alone twelve times (15 s duration) at variable intervals (mean: 4.2 min; minimum: 2 min) over a 54 min period. On later days, the levers retracted after 20 s to avoid contaminating the measurement period with anticipation of retraction of the lever. On day 6, three 10 s tones were also presented at variable times, once in every third of the session (mean interval: 17.5 min).

### 2.4. Measurement and analysis

The conditioning chambers automatically recorded whether, in each 0.1 s interval, the subject was in contact with the lever, nosing the food trough, and/or on the platform immediately before the food trough. These counts were collected in 15 s bins, yielding a value of 0–150 for each dependent variable for each 15 s period measured. During the test phase, response to the lever was assessed by measurement of twelve lever insertions per day. Baseline responding was assessed by measurement of 12 of 15 s periods, at quasi-randomly selected moments throughout the testing sessions, no less than 1 min after and 2 min before any presentation. For analyses, observations were binned in adjacent pairs. Responding to tone presentations on the first day of extinction was charted for comparison, but not included in analyses (during-tone data were measured in 10 s periods, but scaled by 1.5 to expedite comparison).

Repeated-measures analyses of variance were conducted on the three dependent variables. For the goal-oriented variables (nosing the food trough, and presence on the feeder platform), effects of group, stimulus condition (lever presentation versus baseline), within- and between-day order were tested (full-factorial design). Autoregressive covariance structure was assumed, in recognition of the potential for progressive changes over the course of extinction. Group differences in lever contact were tested similarly, except only for periods of lever presentation, because the lever was retracted in other periods.

## 3. Results

By the conditionality hypothesis, there should be no group differences in response to the lever. Striking group differences were observed in multiple dependent measures. Thus, these results speak clearly against the conditionality hypothesis. All reported data are available from the Dryad digital repository [36].

**Figure 2.**
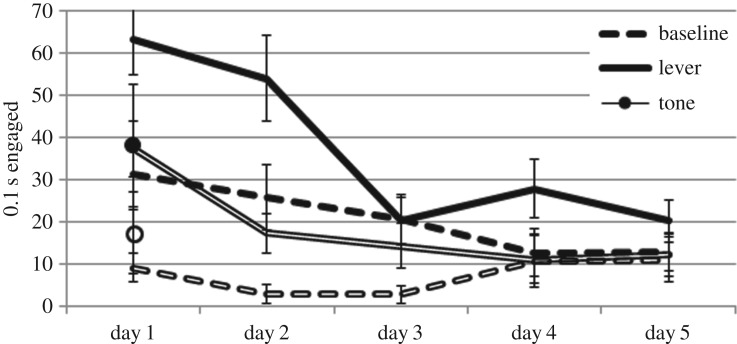
Presence on the feeder platform over the 5 days of extinction during lever presentations, tone presentations (first day only), and baseline recordings for group 75%+ (hollow lines) and group 25%+ (filled lines). Bars show s.e.m.

Subjects were on the feeder platform during lever insertions more than baseline periods (*F* = 41.80, *p* < 0.001) indicating excitatory learning about the lever (figure 2). No effects were observed in nosing of the food trough (all *F*s < 1.5), although note that this was generally low (at floor levels) throughout the extinction sessions (total average: 0.15 s per 15 s measured). Post hoc tests of whether responding to tone presentations was greater than baseline measurements on the first day of extinction revealed no effects for any dependent measure, giving no evidence of learning about the tone.

**Figure 3.**
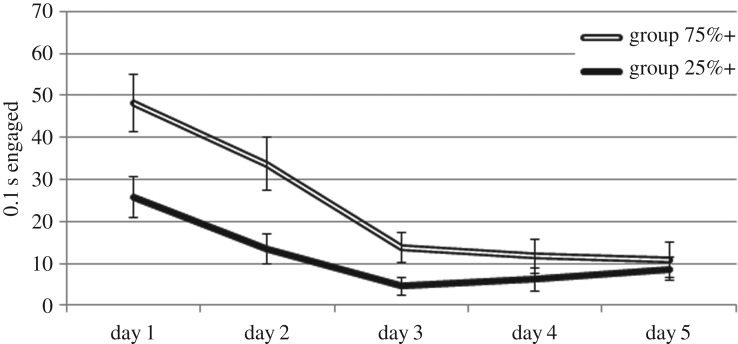
Lever contact during lever presentations over the five days of extinction. Bars show s.e.m.

Striking group differences were observed in presence on the feeder platform (figure 2). In particular, group 25%+ kept closer to the feeder (*F* = 19.97, *p* < 0.001). This was the case not only during lever insertions, but in baseline periods as well, as indicated by no group × stimulus condition interaction (*F* = 1.69, *p* = 0.20), suggesting that group 25%+ subjects generally kept closer to the food trough throughout the session. By contrast, group 75%+ showed greater lever contact during lever insertions than group 25%+ (*F* = 9.29, *p* = 0.004; figure 3). These group differences together indicate that the lever did not carry the same significance to subjects in the two conditions.

The absence of interaction effects of stimulus and group over days (*p*s > 0.3) suggests that the change of response levels by stimulus–condition over days was similar in the two groups. This indicates that both training conditions produced similar patterns of extinction.

## 4. Discussion

The present work identifies and tests a conditionality hypothesis, by which an animal will learn to directly associate not every pair of concurrent stimuli, but rather only those evincing immediate relevance, capturing indirect relevance with second-order associations that are conditional on intervening events. The experiment presents rats with a discernible correlation between insertion of a lever and food delivery that was consistently mediated by another event (CS1). If subjects treat food delivery as conditionally independent of lever presentation, conditional on its immediate antecedent (CS1), the value of the lever as a predictor of food depends exclusively on the second-order path: the extent to which the lever predicts CS1 and the extent to which CS1 predicts food. By this hypothesis, the lever can be a stronger or weaker correlate of food without affecting what the rat learns about it.

In the experiment, although both groups received the same number of lever–CS1 pairings and the same number of CS1–food pairings, groups differed markedly in the extent that lever and food covaried. By the conditionality hypothesis, both groups should have learnt the same lesson, that the lever predicts CS1, and CS1 predicts food, and we should expect no difference between groups in response to the lever thereafter presented alone. By contrast, if subjects learnt to directly associate the lever with food, responding to the lever alone should be greater among subjects for whom the lever had more consistently predicted food delivery. The latter was observed, counter to the conditionality hypothesis.

The absence of conditionality in the older associative learning tradition with roots in Pavlov and Hume, and its apparent ubiquity among graphical model-based views, tempt the erroneous supposition that our experiment presents a contest between these two broad categories of theory. However, both traditions have emphasized reliance on statistical information in learning, and typical graphical model-based theories are inclined to join the older-style association strength-based theories in predicting that our subjects should be moved by the statistical evidence that CS2 and food are dependent, and form a direct, first-order association between them [20,34]. Such theories obviously suffer no empirical embarrassment by the failure of the conditionality hypothesis in the present study. Conceptually, however, our results present no victory to theories that predict them trivially, either by exclusive consideration of statistical information, or by failing to specify the conditions under which animals are expected to acquire conditional associations. Among views that expect animals to show readiness to acquire tidier network structures that preserve conditional independence of select events when circumstances permit, our results present a challenge to articulate how decisions are made amid conflicting information involving multiple kinds of evidence. Few contemporary theories predict our results while meeting this desideratum [20].

Our results are specifically inconsistent with SELT, which predicts that subjects will separately represent the CS2–CS1 and CS1–food associations, and not represent a first-order CS2–food association. Thus, SELT predicts the conditionality hypothesis to hold, and should therefore expect both groups to respond similarly to lever insertions in the extinction phase. SELT may try to account for the observed results by positing that the CS2–food association will be noted eventually, though not at first. This is how its proponents have responded to the challenge presented by the standard conditioned inhibition procedure [15], which otherwise appears to contradict SELT, because a stimulus acquires inhibitory status by presenting a relationship not with the US, but with a second CS that is separately paired with the US [22].

The difference in learning between groups in the present experiment appeared to be qualitative. Subjects in the group for which the lever was a better predictor of food (group 75%+) responded to insertion of the lever in the test phase with greater lever contact. Meanwhile, group 25%+ spent greater time by the food trough. Approach to the food location is the more standard measure of experience-dependent responding to predictors of food, a goal-tracking response. By contrast, contacting a food-predictive lever, in lieu of the goal, is a sign-tracking response [37]. Both groups showed experience-dependent responding to the lever, but the form of the CR was different, with sign-tracking greater in group 75%+ subjects, and goal-tracking greater in group 25%+ (cf. [24]). Thus, it is not necessarily accurate to say that experience-dependent responding was greater in group 75%+. Although the terms of the hypothesis were quantitative, the qualitative group difference suffices to reject the hypothesis.

The greater goal-tracking in group 25%+ is, however, difficult to accommodate for either association strength-based or graphical model-based theories. However, it accords with a third category of theory, behaviour systems [5,6]. By such views, the form of the CR depends on the significance the CS acquires to a system of behaviour or motivation, which is related to such factors as temporal or spatial distance from the US, reliability of the CS–US relationship, motivational significance of the US, and manner of response the CS affords. In the present case, the lever readily affords grabbing and biting, expressions of consummatory motivation, but it can also elicit approach to the food trough, a focal search response. These are the two dependent variables in focus, the sign-tracking and goal-tracking responses, respectively. Which response manifests depends on multiple factors. A consummatory CR is most likely when the CS is proximate and reliable; reducing either factor increases likelihood of acquiring a focal search CR [6,24]. Thus, in group 25%+, for which a tangible but modest CS2 : food correlation was presented, a hearty focal search CR was acquired, whereas the much stronger correlation present for group 75%+ led to a consummatory CR. Although the temporal and spatial distances between CS2 and food were the same for both groups, the conceptual unity of various measures of proximity [38] invites consideration of a network theoretic measure of proximity, number of intervening links in the graph, as a potentially appropriate way of conceiving distance for these purposes. It is feasible that the groups acquired different network structures: group 25%+ subjects may have acquired the conditionality model (as in figure 1*b*), whereas group 75%+ acquired the direct relevance model (figure 1*a*), in which case, the network distance would be greater in group 25%+. Such potential use of network formalism may provide a theoretical connection between CS–US delay and CS–US reliability, the former of which is better supported conceptually, and so a potential point of cross-community borrowing.

Although our results are congruent with previous research [19], extrapolation beyond the specific circumstances of the experiment would be premature. Feeding may show patterns unlike other systems. The particular stimuli selected will have bearing on the results obtained, and the moving BB stimulus used in the current experiment was developed [39] to elicit specifically predatory response patterns, which may be structured differently than non-predatory feeding. Other systems or stimuli that afford different responses may yield different results [6].

The present results put pressure on theories that take a stance on the question of conditionality, and challenge the rest to do so. A theory that expects animals to acquire conditional associations needs to explain why we did not find it in the present experiment. Theories that are silent on the question should break their silence. The question of whether learners represent conditional relevance or not is central not only to graphical model-based theories, but in general, to theories concerning the structure of learning. Whatever one's view of learning, let it answer these questions: should animals acquire conditional associations or not, and why; if so, how, and under what circumstances?

In closing, it is worth stressing that our results do not entitle claims about whether or not rats reason about cause and effect. Rather, they entitle only the mundane conclusion that theories which expect conditionality appear not to capture the behaviour of rats under the presented circumstances. The sophistication at stake in this and related experiments is not the rats', but the theories'.

## Footnotes

1 This use of ‘conditional’ (to mean dependent on specific occurrences) is different in sense from its more frequent usage (to mean dependent on experience), as in the phrase ‘conditional stimulus’, also used in this report by necessity. *Caveat lector*. To help the reader discern these two meanings, we have temporarily adopted the alternative convention of using ‘conditioned’ and ‘unconditioned’ to indicate the latter sense, and have relied on abbreviations (CS, US, CR) more than we otherwise would.

2 ‘Second-order conditioning’ may refer to a training procedure [12], or to a kind of learning that such procedures, among others, might produce [13]. Here, we are primarily concerned with the latter.

## Data accessibility

All reported data can be accessed from the Dryad digital repository: http://dx.doi.org/10.5061/dryad.9b458 [36].
